# In-air and in-water performance comparison of Passive Gamma Emission Tomography with activated Co-60 rods

**DOI:** 10.1038/s41598-023-42978-2

**Published:** 2023-09-27

**Authors:** Riina Virta, Tatiana A. Bubba, Mikael Moring, Samuli Siltanen, Tapani Honkamaa, Peter Dendooven

**Affiliations:** 1 Helsinki Institute of Physics, University of Helsinki, Helsinki, Finland; 2 Radiation and Nuclear Safety Authority (STUK), Vantaa, Finland; 3 Department of Mathematical Sciences, University of Bath, Bath, UK; 4 Department of Mathematics and Statistics, University of Helsinki, Helsinki, Finland

**Keywords:** Nuclear fuel, Experimental nuclear physics, Nuclear waste, Applied mathematics, Imaging techniques

## Abstract

A first-of-a-kind geological repository for spent nuclear fuel is being built in Finland and will soon start operations. To make sure all nuclear material stays in peaceful use, the fuel is measured with two complementary non-destructive methods to verify the integrity and the fissile content of the fuel prior to disposal. For pin-wise identification of active fuel material, a Passive Gamma Emission Tomography (PGET) device is used. Gamma radiation emitted by the fuel is assayed from 360 angles around the assembly with highly collimated CdZnTe detectors, and a 2D cross-sectional image is reconstructed from the data. At the encapsulation plant in Finland, there will be the possibility to measure in air. Since the performance of the method has only been studied in water, measurements with mock-up fuel were conducted at the Atominstitut in Vienna, Austria. Four different arrangements of activated Co-60 rods, steel rods and empty positions were investigated both in air and in water to confirm the functionality of the method. The measurement medium was not observed to affect the ability of the method to distinguish modified rod positions from filled rod positions. More extended conclusions about the method performance with real spent nuclear fuel cannot be drawn from the mock-up studies, since the gamma energies, activities, material attenuations and assembly dimensions are different, but full-scale measurements with spent nuclear fuel are planned for 2023.

## Introduction

Finland will soon be the first country in the world to start disposal of spent nuclear fuel. The Finnish geological repository for spent nuclear fuel is currently being built at Olkiluoto, Eurajoki, and will go into operational phase around 2025. This first-of-a-kind facility consists of an encapsulation plant and a deep underground repository. Nuclear fuel that has been used in the reactor is first cooled in reactor ponds for a few years and then transferred to interim storage pools for at least 20 years of further cooling. From the storage, fuel items are transferred to the encapsulation plant for placement in disposal canisters made of copper and iron. The canisters will then be placed in deposition holes excavated 430 meters below ground level in the bedrock, where they are destined to rest for hundreds of thousands of years^1^.

To make credible safeguards conclusions about the fuel being disposed of, all items need to be verified with non-destructive assay (NDA) methods prior to disposal. Two methods will be used for this, namely Passive Gamma Emission Tomography (PGET)^2^ to verify the integrity of the nuclear fuel, and Passive Neutron Albedo Reactivity (PNAR)^3,4^ to verify the fissile content of the fuel. The main safeguards measurements will be conducted under water at the spent nuclear fuel storage ponds, but there will be a possibility to repeat the measurements at the encapsulation plant. This measurement would take place in air.

The PGET method has been studied extensively throughout the recent years, both with the help of simulated data^5–7^ as well as with measured data from spent nuclear fuel^8–10^. The IAEA has previously released a set of PGET data from measurements of irradiated Co-60 rods, and this dataset has also been used widely^6,11–13^. The research on PGET data analysis and image reconstruction methods has led to major improvements in image quality and helped in building trust on the method for safeguards verification of spent nuclear fuel. Other similar tomographic devices have been developed around the world, for example the YSECT device in Korea^14^ as well as the PLUTO device in Sweden^15,16^.

Despite the vast research conducted worldwide, the performance of the PGET device has never been tested with air as the medium. Our current studies investigate whether the measurements behave as expected when the measurement medium is changed. The in-air performance was tested at the Atominstitut in Vienna, Austria, with a unique measurement setup consisting of activated Co-60 rods, mimicking the dimensions and layout of real light water reactor nuclear fuel.

## Materials and methods

### PGET device

Non-destructive methods to assay spent nuclear fuel are essential from the nuclear safeguards point of view to gain information about the fuel items that are highly active and contain large amounts of special nuclear materials. A highly accurate and efficient method to assay the fuel is Passive Gamma Emission Tomography (PGET), which provides even rod-level detection of missing fuel rods in the 2D cross-section of the fuel^8–10^.

**Figure 1:**
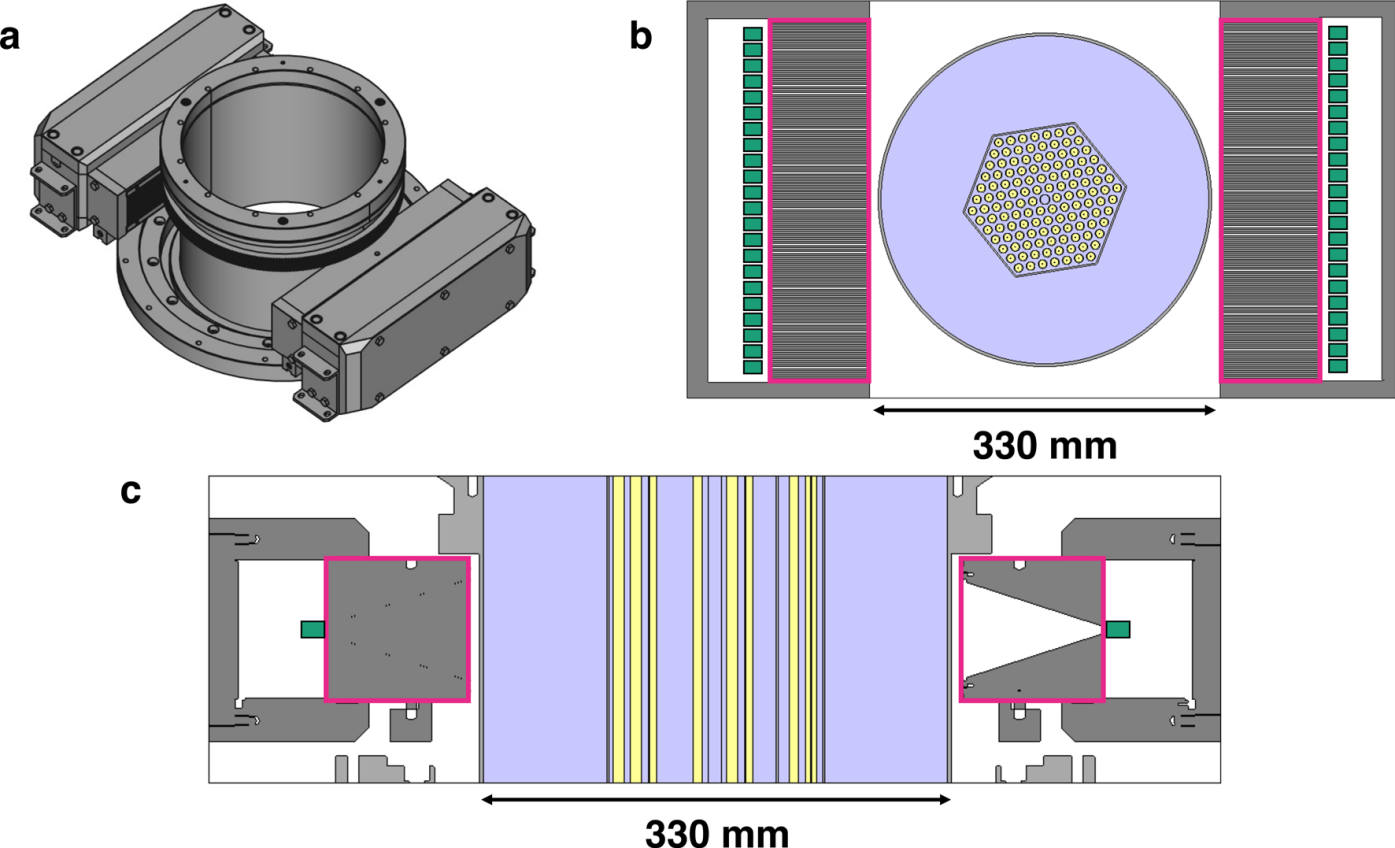
Technical drawing of the PGET device. (**a**) Device structure without the outer cover. Detector banks are visible on both sides of the central hole. (**b**) Transaxial cross section, showing the collimators on opposite sides of the device and the fuel assembly in the central hole. Collimators are circled with pink, detectors in green, water in light violet, fuel in yellow and tungsten shielding in dark grey. (**c**) Vertical cross section, illustrating the tapered collimator and the detector positioning with respect to the measured fuel assembly.

Figure 1 shows a simplified schematic figure of the PGET device. The torus-shaped housing of the device holds two gamma detector banks on opposite sides. The 3.5 mm $\times$ 3.5 mm $\times$ 1.75 mm CdZnTe semiconductor detectors are fixed behind 100 mm thick tungsten collimators with 1.5 mm slit width and a pitch of 4 mm, resulting in a spatial image pixel size of 2 mm $\times$ 2 mm. From all sides except the face of the collimator, the detectors are shielded with at least 30 mm of tungsten. The collimator slits are conical in shape in the axial direction, allowing for shorter data acquisition times. The axial field of view of the device in the center of the torus is around 18 cm. For the experiment described in the present work, 164 detectors were used. Their field of view easily covers the whole imaged object.

The CZT detectors each collect a signal that is first preamplified and shaped and then sent through a discriminator. The discriminator has four thresholds that can be individually chosen for each detector. The power to the electronics module and the data back from the detectors to the control unit are supplied through an ethernet cable. The control unit is connected to a PC, where a dedicated software can be used to choose the measurement parameters, to calibrate the detectors and to provide live count rate and spectral data. The device is described in more detail in earlier publications^2,8,17,18^.

The detector banks are rotated 360 degrees around the spent nuclear fuel assembly, which is placed inside the central hole of the torus. The distance from the center of the measured fuel assembly to the face of the collimator is around 18 cm. The measurements are usually conducted underwater and in the case of spent nuclear fuel imaging last around 5 min. In the work described here, the measurements were done with activated Co-60 rods, which have a much lower activity than real spent nuclear fuel, and thus the measurement times were of the order of hours, not minutes. The detector banks stop at each measurement angle for a pre-defined period of time to gather a sufficient amount of data before the acquisition angle is again changed.

### Measurement setup with activated Co-60 rods

The International Atomic Energy Agency (IAEA) has a test facility at the Atominstitut in Vienna, Austria, where PGET measurements can be conducted with Co-60 mock-up fuel rods in a shallow pool. Natural cobalt rods have been activated at the TRIGA-II research reactor in 2017, resulting in accumulation of Co-60. The rods can be classified in five groups with slightly different activities, due to the positioning of the rods in the reactor core during irradiation. The differences between the activity groups (max 15%) were judged to be relatively small and thus effectively negligible. At the time of the measurements, the mean activities were estimated to be from 3.98 to 4.42 MBq.

The activated Co-60 rods have a diameter of 7 mm and are enclosed in aluminum shells with an outer diameter of 10 mm and a thickness of 0.75 mm. The inactive steel rods are plain steel with a diameter of 8 mm. All rods are 10 cm tall, and thus fully visible in the axial field of view of the PGET device.

The facility is equipped for in-water measurements with the activated Co-60 rods and has multiple grids where the rods can be arranged in a desired order. For the measurements, a VVER-440 grid was chosen based on the previously encountered difficulties in distinguishing the central parts of this type of fuel^9^. Although the disposal of spent nuclear fuel in Finland will be starting with the BWR fuel, this VVER type is important to investigate since it poses more difficulties for the image reconstructions due to the denser packing of the fuel rods.

For the in-air measurements, a setup was built to allow for the Co-60 grid to be measured both in air and in water, without having to change the placement of the grid. Measurements were conducted both in air as well as in water to be able to fully compare the performance of the PGET device with respect to the medium around and in between the measured Co-60 rods.

#### In-air setup

**Figure 2:**
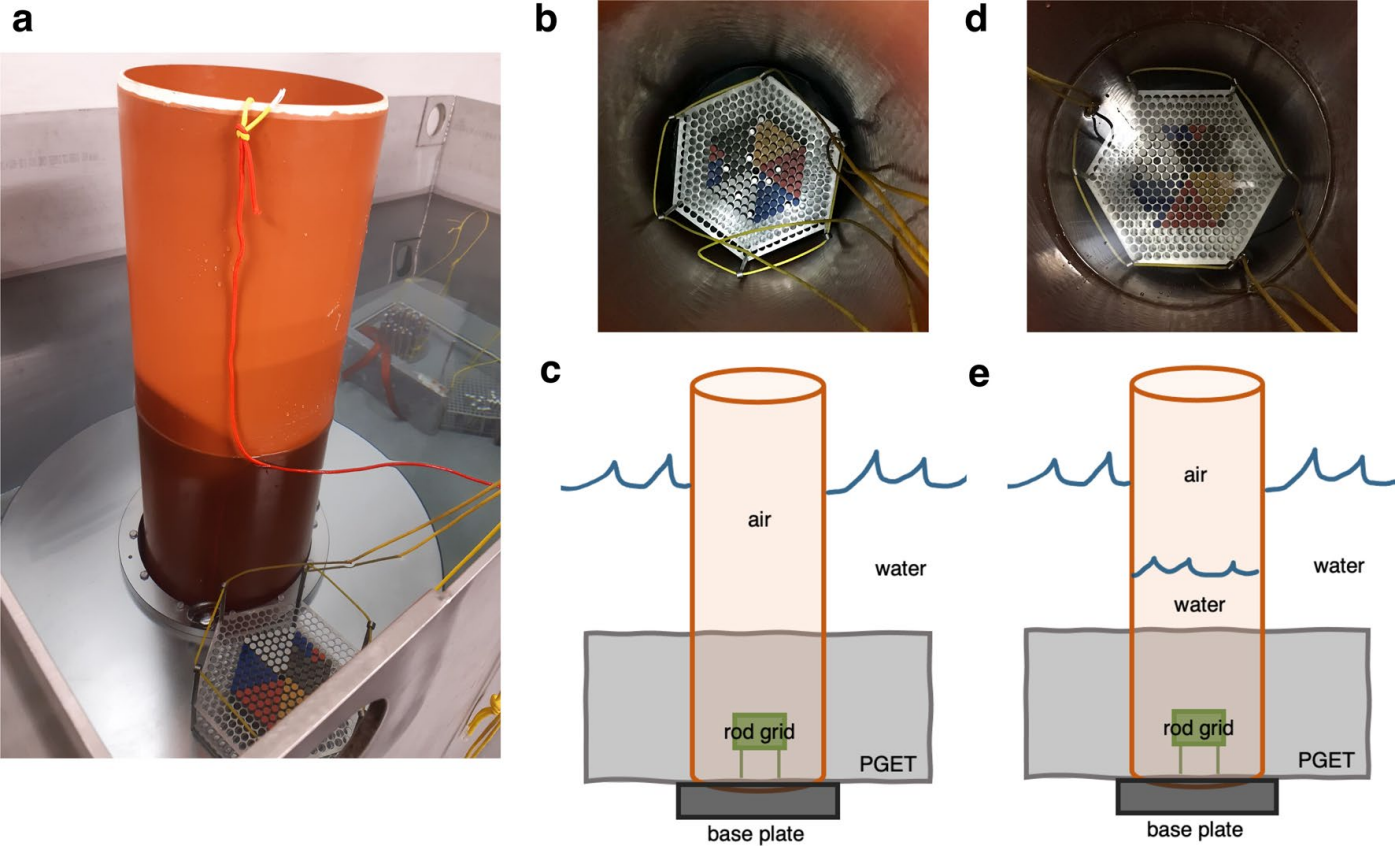
Measurement setups. (**a**) Side view of the setup submerged in the measurement pool. The Co-60 rod grid is resting on top of the PGET outer cover before placement inside the tube. (**b**) Top view of the in-air setup tube with the grid in place for measuring. (**c**) A schematic illustration of the in-air measurement setup from the side. (**d**) Top view of the in-water setup tube with the grid in place for measuring submerged under water. (**e**) A schematic illustration of the in-water measurement setup from the side. Notice the water level inside the tube.

Figure 2 shows the main components of the measurement setup from different sides. The custom-built setup consists of a 31.5 cm diameter hollow T-shaped polyethylene pipe with a wall thickness of 8 mm and a heavy steel base plate at the bottom. The pipe is fitted tightly inside the PGET central hole from below, so that the top of the pipe extends above the water surface. The base plate fixes the pipe into place and the weight of the PGET device compensates for the buoyancy of the air volume inside the pipe. When the setup is submerged under water, the opening at the top allows for easy access to the Co-60 rods for changing between grid layouts.

#### In-water setup

For the control measurements in water, the pipe is filled with water until the measurement grid is fully submerged under water (see Fig. 2d,e). Thus, the only thing changing between the air and water measurements is the measurement medium. The placement of the Co-60 rod grid and the slight effect of the polyethylene wall of the pipe are the same for both measurements.

#### Measured Co-60 grids

**Figure 3:**
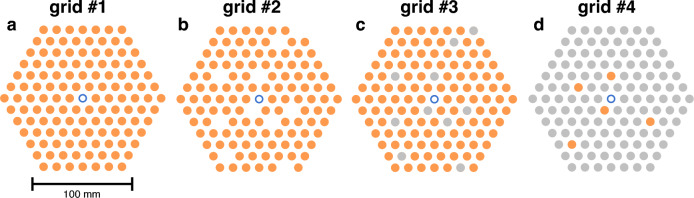
Measured Co-60 grids. Orange circles denote activated cobalt rods, grey circles denote inactive steel rods and blue open circles denote central water positions. (**a**) Grid #1, full grid with 126 activated Co-60 rods. (**b**) Grid #2 with 10 rods removed. (**c**) Grid #3 with 10 rod positions with inactive steel rods (grey). (**d**) Grid #4 with 5 activated cobalt rods (orange) and 121 inactive steel rods (grey). Note that the positions of the modified rods in grids #2 and #3 are identical.

Four different Co-60 grid layouts are used in the investigations. Figure 3 shows schematics of the grids, where the rod positions are arranged in a hexagonal manner, simulating a VVER-440 fuel grid. The rods are arranged in a hexagonal basket which has a top and a bottom plate with 10 mm holes and the rod positions are separated with a 12.2 mm pitch. This corresponds to the dimensions of the real fuel assemblies used in VVER-440 nuclear reactors.

First, a grid fully filled with activated rods is measured, then 10 of these rods are removed and next the same 10 rod positions are filled with inactive steel rods. The fourth layout consists of only 5 activated Co-60 rods and the rest of the grid is filled with inactive steel rods. Table 1 shows information about the number of different kinds of rods in each grid. The results shown in this work focus on grids #2 and #3, and results from grid #4 are discussed in detail in a separate section. The grid #1 results are not further discussed since the grid arrangement is the simplest and the results provide no additional information.

**Table 1:** Number of different rods in the measured Co-60 grids.

Setup number	Activated rods (Co-60)	Empty positions	Inactive rods (steel)	Water channel positions
#1	126	–	–	1
#2	116	10	–	1
#3	116	–	10	1
#4	5	–	121	1

### Measurement parameters

Most of the measurements were done with 360 data acquisition angles (1-degree steps) and with a data acquisition time of 62422 ms per angle, resulting in a total data acquisition time of around 6 h 15 min. For each angle, the rotating detector banks stop for data acquisition. This differs from the standard continuous mode of operation for the detector bank rotation, but is needed here due to the very long measurement times at each angle to get sufficient counting statistics from the activated Co-60 rods. The chosen measurement time was a compromise between counting statistics and the limited time available for the measurement campaign at the facility.

For some of the measured setups, two consecutive measurements were made without changing anything in the grid. These back-to-back measurements allow for a comparison of the setup counting statistics between runs and show how large the variation can be with these low activities. For some analyses of the results, these consecutive measurements are added up to simulate an even longer measurement of 124844 ms per projection angle. This summation is possible because the data are acquired at identical positions and the setup is not otherwise altered between measurement rounds in these cases.

**Figure 4:**
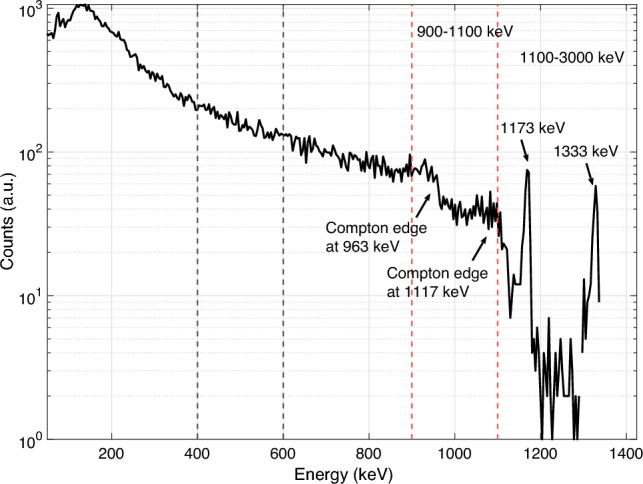
Gamma energy spectrum of a Co-60 assembly. The energy windows of data acquisition are shown with dashed lines. The full energy peaks of Co-60 are denoted along with the Compton edges of these peaks.

Data were acquired in four gamma energy windows. The measured isotope Co-60 energy spectrum has two full energy gamma peaks, at 1173 keV and at 1333  keV, and corresponding Compton edges for the peaks at 963 keV and at 1117 keV. For the analysis presented here, the energy windows capturing these characteristics are used, namely the window capturing most of the Compton edges (900–1100 keV), and the window capturing the photopeaks themselves (1100–3000 keV), or the sum of these two. Figure 4 shows a gamma energy spectrum from a similar but larger Co-60 assembly than was studied in this work. Around 30 min were spent at each measurement angle to produce enough counting statistics for the gamma characteristics to be well visible. This measurement was conducted with the same PGET device with water as the measurement medium. 512 gamma channels were used for this measurement, each approximately 4 keV wide. The energy resolution of the CdZnTe-semiconductor detectors used in the PGET device is 10.7 ± 0.9 keV FWHM (full width at half maximum) at 1333 keV.

### Compton edge

The most prominent interaction mechanism of photons with matter at the studied 1 MeV neighborhood is the Compton effect^19^. For the tomographic image that is reconstructed from the data, photons that travel unscattered from the imaged object are valuable. Usually, the gamma energy acquisition windows are tuned to capture the full energy peaks of the isotope, but our investigations have shown that the Compton continuum can prove to be equally valuable^10^.

The Compton continuum is formed by the photons that arrive unscattered at the detector, but scatter in the detector itself. The energy that is released to the detector in the Compton scattering depends on the scattering angle. The Compton edge is the highest-energy scattering for the scattered electron that corresponds to 180 degree angle. By capturing this part of the gamma energy spectrum in the measurement window, we gain all the photons that do not scatter until the detector, and thus still carry the maximum directional imaging information. The data in this window are highly valuable in terms of good directional information about the imaged object, and even in some cases comparable to the data from the full energy peaks themselves.

The energy of the scattered electron in Compton scattering can be derived from the conservation of energy and momentum^19^, and is

1 $$\begin{aligned} E_{e^{-}} = E_{\text {incident}} \biggl ( 1 - \frac{1}{1+\frac{E_{\text {incident}}}{m_0 c^2}(1-\cos \theta )} \biggr ), \end{aligned}$$

where $E_{\text {incident}}$ is the energy of the incident photon (in this case either 1333 keV or 1173 keV), $m_0 c^2$ is the rest-mass energy of the electron (0.511 MeV) and $\theta$ is the scattering angle of the photon. For the case of the Compton edge, the scattering angle is 180 degrees. The energy absorbed by the medium is the energy of the scattered electron.

In this study we used a gamma energy window of 900-1100 keV to capture most of the two Compton continuums originating from the Co-60 full energy peaks.

### Image reconstruction

The process of computing reconstructed tomographic images from the measurement data is described in detail in earlier publications^9,20,21^, but is briefly summarized here.

The raw gamma data obtained from the measurements are preprocessed to account for the differences in the detector sensitivities and for the dead times of the detectors. Based on an initial filtered back-projection (FBP) image, a grid of rod positions is fitted on the image and these positions are used as a geometry prior for an iterative reconstruction algorithm. The task is formulated as a constrained minimization problem with a data fit term and regularization terms. The activity and the attenuation image of the object are simultaneously computed. Bounds are applied to the attenuation and activity values to further limit the space of possible solutions. The geometry prior causes the reconstruction to favor solutions which have rod-shaped objects at the predefined positions, but nothing is assumed of the contents of these circular items.

### Image quality index

The so-called “image quality index” is a numerical tool to help in assessing the goodness of the reconstructions in detecting empty grid positions inside the fuel grid. It was introduced in an earlier publication by Virta et al.^10^, where it is explained in more detail. The main purpose of this index is to evaluate the quality of the reconstructed images in the context of the geological repository safeguards. Therefore, the ability of the method to detect missing fuel rods is essential, and this is the quality that the image quality index assesses.

The image quality index is a pair of two values, $[\Delta ,\sigma _f]$, which describe how well the group of inactive grid positions is separated from the group of active grid positions. The value $\Delta$ is defined as $\Delta \equiv (\mu _f - \sigma _f) - (\mu _e + \sigma _e )$, where $\mu _f$ and $\mu _e$ are the means and $\sigma _f$ and $\sigma _e$ are the standard deviations of the average activity of the active grid positions (filled positions) and inactive grid positions (usually empty), respectively. These two values allow for quantitative investigation and comparison of results acquired in different conditions and with different parameters.

## Results and discussion

### Comparing results from the Compton edge and the photopeak energy windows

**Figure 5:**
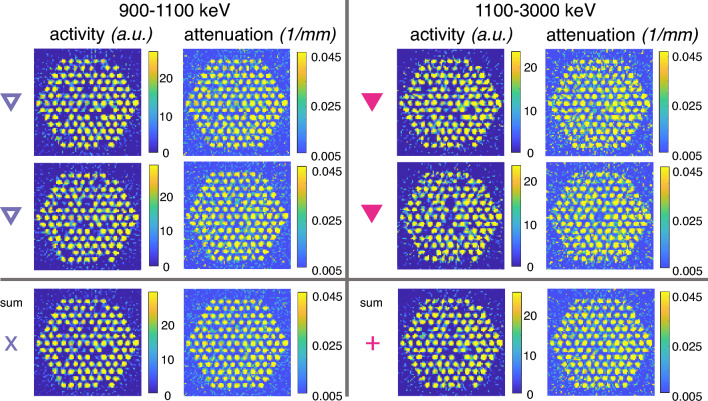
Activity and attenuation reconstructions for grid#3 with water as the measurement medium. The data on the left are from a gamma energy window of 900–1100 keV, and the data on the right from a gamma energy window of 1100–3000 keV. Activity values are normalized to the highest value in each subfigure. The attenuation value minima are set to the water linear attenuation coefficient, and the maximum is the linear attenuation coefficient of cobalt, for a gamma energy of 1253 keV (mean of the two Co-60 photopeak energies). The last row shows the two above measurement data summed together for an effective longer measurement time. Symbols link the measurement cases to the symbols in Fig. 6.

Reconstructed images from the photopeak energy window 1100–3000 keV were compared with images from the Compton edge energy window 900–1100 keV to study the feasibility of using the Compton continuum for high-quality images. Figure 5 shows these reconstructed activity and attenuation images for grid #3 measured in water.

**Figure 6:**
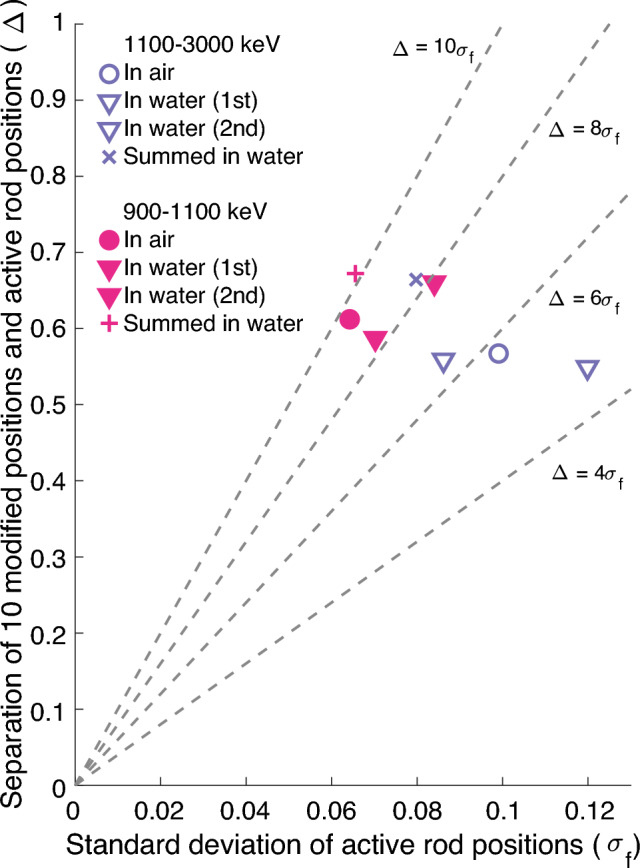
Image quality index plot for comparisons between different gamma energy windows. The dashed lines represent different acceptance criteria in terms of constant $\Delta /\sigma _f$. Results from the photopeak gamma energy window (1100–3000 keV) are denoted with violet (open marker) and results from the Compton edge window (900–1100 keV) with pink (filled marker). All measurements were done with grid #3, both in air as well as water. “Summed” datapoints are a combination of identical back-to-back measurements, denoted with similar symbols in the figure. The data are provided in supplementary material online.

Figure 6 shows the image quality index plot for grid #3 in the two different gamma energy windows, both in air as well as in water. The separation of the two different groups ($\Delta$) used in the image quality index calculation was computed with the 10 modified positions, which in the case of grid #3 are the substituted positions. Overall, results from the 900–1100 keV window show better separation of the inactive and active rod positions than the results from the 1100–3000 keV window. In terms of $\Delta /\sigma _f$, the values are around 8–10 for the 900–1100 keV window and around 5–7 for the 1100–3000 keV window.

This result is a bit unexpected since the photopeak, which carries the most direct imaging information from the measured item to the detectors, is captured in the 1100–3000 keV window, which here shows slightly worse image quality. However, the low counting statistics of the measurements affect this result heavily. On average, the detectors that see the fuel get around 62 counts per sinogram pixel (average for the detectors that see the fuel) for the 900–1100 keV window with air as the medium, whereas for the 1100–3000 keV window this number is only 24. In water the lower energy window gets around 36 counts per sinogram pixel and the higher energy window only 14. Since there are more detected counts in the 900–1100 keV window with the Compton edges, the results are better. The difference is not very large but noticeable.

It is also clear that the summed data from back-to-back measurements result in notably better separation between the two rod groups. For the 1100–3000 keV window in-water measurements, the individual measurements result in a $\Delta /\sigma _f$ ratio of around 5–7, whereas the summed-up results are over 8. For the in-water measurements of the 900–1100 keV window, the individual measurements have values of around 8 and the summed-up data of around 10. This also backs up the notion that the low counting statistics have a high effect on all of the presented results.

### Comparison of in-water and in-air performance

For a more thorough comparison of in-water and in-air performance, the following analysis was conducted by combining the two energy windows 900–1100 keV and 1100–3000 keV, to minimize the effects of the low counting statistics.

**Figure 7:**
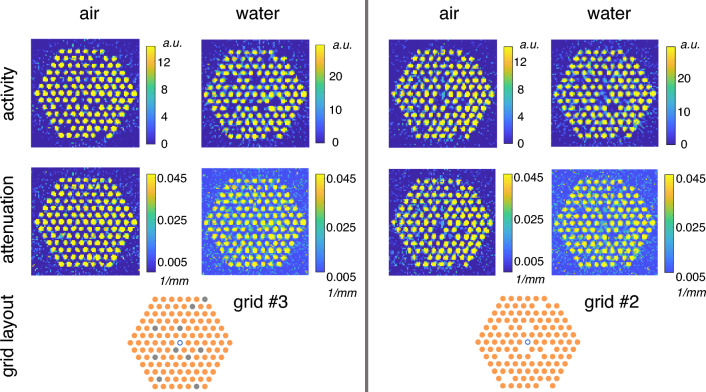
Activity and attenuation reconstructions for grids #2 and #3, with air and water as the measurement medium. All data are from a combined gamma energy window of 900–3000 keV. Activity values are normalized to the highest value in each subfigure. The attenuation value minima are the air and water linear attenuation coefficients, respectively, and the maximum is the linear attenuation coefficient of cobalt, for a gamma energy of 1253 keV (mean of the two Co-60 photopeak energies). The activity and attenuation values are imposed as boundaries in the minimization problem. In the grid layout images, orange circles represent activated rods and grey circles represent inactive steel rods.

Figure 7 shows the reconstructed activity and attenuation images for the Co-60 grids #2 and #3, both in air as well as in water, calculated for a combined gamma energy window of 900–3000 keV. No drastic differences between the images are apparent to the naked eye. For grid #3, the inactive steel rods appear as empty positions in the activity image but are quite similar to the active rods in terms of attenuation. For grid #2, the empty positions appear in both the activity and attenuation images, as expected. For all reconstructed images, the central water position is not very well visible due to the high attenuation in the fuel grid.

**Figure 8:**
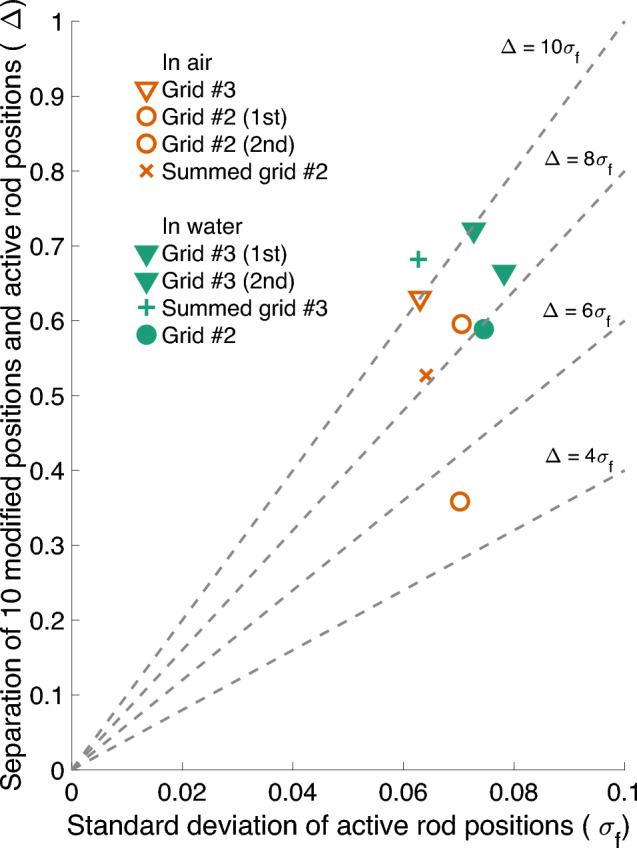
Image quality index plot for comparisons between performance in different media. The dashed lines represent different acceptance criteria in terms of constant $\Delta /\sigma _f$. Comparison between in-air (orange open marker) and in-water (green filled marker) results of measurement setup grids #2 and #3 (“10 removed” and “10 replaced”, respectively), are plotted from a combined gamma energy window of 900–3000 keV. “Summed” points correspond to the sum of two individual back-to-back measurements from the same setup. The data are provided in supplementary material online.

Figure 8 shows the image quality indices for grids #3 and #2, measured both in air and in water. The separation of the two different groups ($\Delta$) used in the image quality index calculation was computed with the 10 modified positions: empty positions for grid #2 and substituted positions for grid #3.

Overall, the measurements conducted in water seem to to have a bit better separation between the empty or substituted versus the active rod positions. In terms of $\Delta /\sigma _f$, for the measurements in water the indices are around 8–10, whereas for the air measurements the values are around 5–8, with the exception of the measurement of grid #3 in air, which has a value of around 10.

Despite the small differences in the image qualities, it is evident that due to the low number of counts recorded during the measurement, the variation even between back-to-back measurements is quite large. For grid #2, the difference in $\Delta /\sigma _f$ for two back-to-back measurements is over 3 (from 5 to 8) and even the summed-up data have only a value of around 8. It is an improvement if you look at the image quality index average of the two consecutive measurements, but not better than the better one of these measurements. For the in-water measurements of grid #3, the difference between the two consecutive measurements is almost 2 in terms of $\Delta /\sigma _f$. The summed-up results show a minor improvement to around 10 for this case.

For the detection of replaced versus removed rods, the results would indicate that the inactive steel rods are easier to detect than empty positions in the measurement grid. However, with such a low number of measurements and the evident effect of the measurement statistics, this could be by accident. To be able to draw further conclusions, the setup would need to be measured multiple times in similar conditions, or a measurement of even longer data acquisition time would need to be conducted.

The linear attenuation coefficients approximated for the mean energy encountered in the experiments (1253 keV) are $\mu _{\text {H}_2\text {O}} = 6.32 \cdot 10^{-3}$ 1/mm for water and $\mu _{\text {steel}} = 4.21 \cdot 10^{-2}$ 1/mm for steel. Such a large difference in attenuation could explain why the replaced rods might be easier to detect than empty grid positions. Similarly, for the comparison of air and water, the difference is also large: water absorbs gamma rays a lot more than air ( $\mu _{\text {H}_2\text {O}} = 6.32 \cdot 10^{-3}$ 1/mm vs. $\mu _{\text {air}} = 6.85 \cdot 10^{-6}$ 1/mm). To make clear conclusions about the mechanism causing the observed differences in rod detection, the photon interactions would need to be studied with detailed simulations at the rod level with different media (steel, water and air).

Overall, all of the reconstructed images show good separation between the different rod positions, since even the worst results have a $\Delta /\sigma _f$ ratio of at least 5. In terms of detecting rod positions correctly, the conclusion is that the measurement medium does not have a crucial effect.

### 5 emitting rods in a grid of attenuating rods

The reconstruction method was further investigated by conducting measurements of a grid where only 5 of the 126 rod positions contained an activated Co-60 rod, and the other positions were filled with inactive steel rods (grid #4).

**Figure 9:**
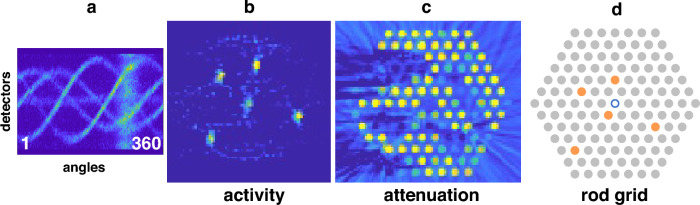
Grid #4 with only 5 active rods in a grid of steel rods, measured in air with the combined gamma energy window of 900-3000 keV. (**a**) Measured sinogram showing the structure of the imaged object. Measurement angles from 1 to 360 are on the x-axis, detectors on the y-axis. (**b**) Reconstructed activity image, clearly indicating the five active rods. (**c**) Reconstructed attenuation image, showing traces of the correct grid but including areas of low attenuation. (**d**) The ground truth placement of the rods in the grid. Grey circles denote inactive steel rods and orange circles denote activated Co-60 rods.

Figure 9a shows the sinogram of such a measurement of grid #4, where the individual emitting rods can clearly be distinguished. The sinogram contains a band of high-activity background at angles between 240 and 310. This was found out to be caused by the rest of the activated Co-60 rods that were placed at the bottom of the pond during the measurements. The large amount of radiation originating from the side was insufficiently attenuated by the 50 cm of water in between, and reached the detectors at certain rotation angles through the face of the collimator slits.

Figure 9b shows the reconstructed activity image for grid #4. The activated rods are clearly reproduced. Figure 9c shows the attenuation reconstruction, which is of poorer quality, partially reproducing the correct grid of rods but including areas of lower attenuation. Figure 9d shows the arrangement of rods in the grid.

The lower-attenuation areas in the attenuation reconstruction are likely caused at least partially by the background at certain angles. The irregularities in the attenuation grid appear at the same angles as the background and seem to extend from the five emitting rods outward in the direction of those angles. The higher activity in the region is interpreted as lower attenuation by the algorithm, which is not equipped for compensating for background at such distinct angles and originating outside the imaged volume. However, to confirm this hypothesis, a no-background measurement or simulation would be needed.

Another aspect affecting the quality of the attenuation reconstruction is the poor statistics of the measurement. The noise in the measurement is assumed to be Gaussian by nature, and the mathematical implementation of the minimization is based on this assumption. The count rates during the data acquisition for this measurement grid are so low that this assumption no longer holds, and rather, the noise follows a Poisson distribution. This is not implemented in the reconstruction process for the results shown here. For the context of geological repository safeguards, such a low activity measurement is irrelevant in practice, and thus the changes in implementation are not considered here.

These measurements with an unusual arrangement of rods allow for a deeper understanding of the measurement method. Continuing with the knowledge gained in simulating quite a similar grid with the reconstruction forward model (as reported in^10^), these results show fundamental aspects of the measurement of grids of highly attenuating rods. The activated Co-60 rod that is closest to the grid center has a very minor contribution to the measured sinogram compared to the rods that are closer to the edges of the assembly. This is a natural consequence of the high attenuation of the steel rods, and is even more pronounced with uranium oxide for the case of real spent nuclear fuel and the lower gamma energies encountered there.

### Extrapolation of results to concern real spent nuclear fuel

The studied mock-up setup does unfortunately not provide results that fully represent the performance of the device with real spent nuclear fuel. The setup has its limitations due to the lower activity and shorter length of the imaged Co-60 rods and the fundamental differences that the different materials and gamma energies create.

This study shows that the measurement medium poses no limitations to the PGET method in the 2D plane that is imaged. This being said, the possible background originating from the full-length nuclear fuel assembly could not be studied with this setup. Under usual measuring conditions under water, the radiation originating from above and below the measurement plane is efficiently attenuated by the water in between the fuel assembly and the detectors. However, when measuring in air, this attenuation is removed and more gamma rays will enter the detectors from unwanted directions, creating background to the useful signal. This is a situation that needs to be studied in more detail with the help of simulations and full-scale measurements with spent nuclear fuel.

The other limitation of the setup is the different materials used. Co-60 emits gamma rays at considerably higher energies than the Cs-137 that is the dominating gamma emitter in aged spent nuclear fuel. The linear attenuation coefficient of cobalt for the Co-60 photopeak energies is $\mu _{\text {Co}} = 4.69 \cdot 10^{-2}$ 1/mm. On the other hand, for uranium oxide at the 662 keV gamma energy of the Cs-137 photopeak, the linear attenuation coefficient is $\mu _{\text {UO}_2} = 0.136$ 1/mm. If we take a gamma ray and assume a worst-case-scenario of 40 mm of rod material to travel through to get to the detectors, using the Beer-Lambert law we can calculate for the mock-up fuel case that around 15 % of the original gamma rays reach the detectors, whereas for the real spent nuclear fuel the amount is only 0.44 %. For the comparison of measurement medium, this should not have a large impact, since the air measurements were always benchmarked with a similar water measurement. However, the inner parts of the grid are easier to image with the mock-up rods than with spent fuel, and thus no further conclusions about the method’s ability in air with real spent nuclear fuel should be drawn based on the Co-60 measurements.

## Conclusion

The performance of the PGET method in different measurement media was evaluated by conducting measurements with activated Co-60 rods in air and water. The results show that the measurement medium around and in between the measured rods does not have a significant effect on the image quality, evaluated in terms of separating rod positions that were filled with Co-60 rods, substituted with an inactive steel rod or completely empty. Measurements in water would seem to show a bit better separation of filled and empty or substituted rod positions, but the counting statistics of the measurements is so low that this conclusion requires further investigations to be verified. Still, all measurements show a good separation between these groups of rod positions, and missing or substituted rods could be identified with high confidence from grid positions including activated rods. The limitations of the measurement setup with activated Co-60 rods need to be addressed with appropriate simulations and full-scale measurements. The feasibility of the method for real spent nuclear fuel measurements in air needs to be investigated further, properly handling the absence of the background-attenuating water and possible scattering of radiation from the room surrounding the measurement position.

### Supplementary Information

Supplementary Information.

## Data Availability

The datasets used and/or analysed during the current study can be made available under a collaboration agreement with the Helsinki Institute of Physics and The Radiation and Nuclear Safety Authority of Finland. Please contact the corresponding author for more information.
